# A University Degree for English Dentists

**Published:** 1903-07-15

**Authors:** Wm. Guy

**Affiliations:** Dean Edinburg Dental Hospital


### A UNIVERSITY DEGREE FOR ENGLISH DENTISTS.

BY WM. GUY, F.R.C.S., L.D.S.
Dean Edinburg Dental Hospital.

I think it does not require an unusual gift of prescience to perceive that the question whether or not our ancient universities should frame curricula and examinations for a Dental Degree is one which, if not imminent, will become so in the not far distant future. There is a pretty general concensus of opinion in the educational world that the universities must, if they are to retain their importance as factors in our national life, shake off their mediæval shackles and come out in to the light prepared to see things as they are to-day, and as they are likely to be to-morrow. They have set out on the path of progress, but they advance tardily and almost unwillingly along it. But, as the genius of the Anglo-Saxon race finds itself specially triumphant in the adaptation of ancient institutions and methods of existing needs and demands, there need be no despair. I doubt not but that the universities will gradually but inevitably assume the more extensive functions which the ever-growing demand for highly specialized education and technical instruction in every branch of science, the arts, manufactures and commerce will impose upon them. It is quite a tenable proposition that the universities ought to have remained to all time places meant only to provide for the instruction of youths in the humanities, and for the maintenance of a limited number of the most distinguished and cultured alumni in comfortable and elegant conditions of lettered ease. In that case they might well have been left to contemplate with ecstacy the glories of the past, and with dismay the headlong course of the present,. But no such attitude of contemplation is now possible. The universities are pledged to progress—*vestigia nulla retrorsum.* "From the primary school to the university" is the popular cry. When the clever primary schoolboy arrives at the university he will expect to find there the best available teaching in whatever he purposes to make the work of his life, be it divinity, arts, law, medicine, gold mining, brewing, shipbuilding, banking, commerce, electrical engineering, or what not. And he will further expect to leave the university with a degree; nor does there appear to be any valid reason why, if he deserves it, he should not get what he desires. The University of London is instituting Boards of Study in a variety of subjects; we hear rumors of a Chair of Commerce in Glasgow, the new University of Birmingham has a Chair of Brewing, it has also a "Department of Dentistry", whatever that may be, Birmingham has, too, the distinction of being the first University in the United Kingdom to grant degrees in dentistry. It is likely, however, that London and the three universities to be formed from the disrupted Victoria University will follow suit, and when this has happened it will be almost incumbent on the Scottish universities to consider whether they should do likewise. When the matter does arise, active steps will hardly be taken without plenty of consideration, it is therefore desirable that there should be a well-informed professional opinion pervading and obtainable from dentists as a body. It is with the view of ascertaining what we think about the question of degrees for dentists and stimulating thought and discussion that I present these remarks.

To bring the subject before you in concrete form I conceive it best first to quote the Regulations for Degrees in Dentistry of the University of Birmingham, then to offer a few remarks on these regulations and finally to indicate the lines on which I think the Scottish universities should proceed, if at any time they should contemplate framing regulations for such degrees.

The Birmingham "Regulations for Degrees in Dentistry" are as follows:

1. "The Degrees conferred by the University, are those of Bachelor and Master of Dental Surgery (B.D.S. and M.D.S.)."

2. "All candidates for these degrees must pass the same matriculation examination as that required from the candidates for medical degrees."

3. "The degree of Bachelor of Dental Surgery in not conferred upon any candidate who has not obtained a license in dental surgery from somebody legally entitled to confer such qualification. The candidate is not eligible for the degree until a period of twelve months has elapsed from the passing of his examination for the license in dental surgery. Of this period, at least six months must be spent in the dental department of a general hospital approved by the university."

4. "In addition to the license in dental surgery, the candidate must produce evidence that he has attended the courses required of the medical students of the university in the following subjects and passed the examination held in the same for medical and surgical degrees: (*a*) Chemistry, and Practical Chemistry; (*b*) Physics, and Practical Physics; (*c*) Comparative Anatomy; (*d*) Anatomy, and Practical Anatomy; (*e*) Physiology, and Practical Physiology. B. That he has attended the following cources: (*f*) One course of lectures on Medicine; (*g*) One course of Lectures on Surgery; (*h*) Special courses of lectures on the Surgery and Medicine of the Mouth; (*i*) Pathology and Bacteriology; And has passed the examinations for candidates for dental degrees held in each of these subjects. C. That he has attended courses in: (*k*) Dental Histology and Patho-Histology; (*l*) Comparative Dental Anatomy; (*m*) Dental Surgery and Prosthetic Dentistry. D. That he has received instructions in the clinical examination of living cases at the dental department of a general hospital for a period of not less than six months."

5. "The final examination will deal with the subjects in classes C and D."

6. "On the expiration of twelve months from the date of passing the examination for the Degree of Bachelor of Dental Surgery—the candidate will be eligible for that of "Master of Dental Surgery."

7. "For this degree candidates will be required to submit a thesis containing original work and investigations in some subject connected with dentistry, which thesis shall be submitted to examiners to be nominated by the Board of Dental Studies. The degree will be awarded or withheld according to the report of these examiners."

These regulations have certainly not been adopted without considerable thought and they may be taken as representing a policy on the success of which it will be impossible for some time to pronounce a verdict, they must be regarded for the present as on their trial. To the course of study prescribed, and the scope of the examination exception can not be taken. The provision that the degree of B.D.S. will not be conferred on anyone till a period of twelve months has elapsed from the passing of his examination for a license in dental surgery is eminently a wise one. But I do not look with so much favor on the regulations that the candidate should pass the examinations held for medical and surgical degrees in chemistry, physics, comparative anatomy, anatomy and physiology. I do not altogether like the notion of the degree being a kind of hybrid one, nor do I care for the way in which the latter part of the curriculum for the degree is tacked on the earlier part of the medical curriculum. I do not wish in any way to emphasize these objections but will rather content myself with indicating the lines on which regulations for degrees in dentistry should in my opinion be framed.

In the first place, I think there is no necessity for any multiplication of degrees. I infinitely prefer the degrees of B.Sc. and D.Sc. to those of B.D.S. and M.D.S., and this perhaps involves my most vital argument. As a guarantee to the public of a man's fitness to practice, the L.D.S. diploma should be all sufficient. If in addition to that he wants a degree, that degree should be an academic distinction, and for my part I would not have it a registrable one, even as an additional qualification, for this is but the first step to obtaining its registration as a primary qualification. Science is all embracing, and it is from the Faculty of Science that a dentist who desires a degree which shall stamp him with the hall-mark of scientific distinction should seek a diploma. If the University of Edinburg ever institutes degrees for dentists I hope those degrees will be the B.Sc. and D.Sc. The arrangement of the curriculum would be a simple one. The first examination should be the same as the first examination for the B.Sc. degree in pure science, *i.e.*, in (i) Mathematics or Biology; (ii) Natural Philosophy; (iii) Chemistry.

The second examination should be on a very high standard in (i) Dental Anatomy, human and comparative; (ii) Dental Histology and Bacteriology; (iii) Dental Surgery and Pathology, and Medicine; (iv) Dental Mechanics and Prosthesis.

No candidate should be admitted to the second B.Sc. until a period of twelve months has elapsed subsequent to his passing the examination for the L.D.S. diploma. He should further be required to produce certificates of having passed that period of twelve months in a recognized dental school, engaged either in special research work, or in demonstrating or teaching some branches of dental science. For the D.Sc. degree the usual course demanding the presentation of a thesis embodying original work or observation should be adopted.

It is vain to expect unanimity on a subject which will appeal to students, dentists, teachers, dental hospital authorities, and university courts in such different ways. But when the time comes for the institution of a degree in Edinburg, I fervently hope that the regulations will be framed on the lines which I have very briefly sketched, and that the Birmingham model will not be copied. The point is this, the degree should be a distinction. Then let it be a scientific distinction. Do not take the student half way to a medical degree and the switch him off on the dental line. If he wants a medical degree, by all means let him go on and take it, he will never regret it. But the greatest boon the dental profession can receive at the hands of the universities is the establishment of a B.Sc. and D.Sc. for dentists.

DISCUSSION.

Dr. Williamson said it was difficult for one outside the active circle of those engaged in dental education to give an opinion on a question of this kind. The reader of the paper had considerable experience in this special education, and was thus fitted to formulate methods of improving the existing conditions.

He was glad to find that Dr. Guy upheld the L.D.S. degree as the vital point in the career of a dentist. Although fully forty years old the public were only beginning to recognize it as the proper hall-mark of qualification, and it seemed to him that as the various medical degrees were a sore puzzle to this same public, it would be very unwise to present them with a similar problem in regard to degrees in dentistry.

In regard to a medical degree, no doubt it was well and good to obtain it; but with the vast increase in medical knowledge and the expanded curriculum designed to cope with that increase it became more of an open question as to whether a great deal of the time thus spent could not be more profitably employed by one whose life work was concerned with organs, parts of a living body, but of which the treatment consisted largely in methods of restoration and repair that were associated more with the various sciences than with those of a strictly med.cal or surgical nature.

Mr. J. S. Amoore, L.D S., said they had from time to time heard a great deal as to a higher qualification for dentists, and they were likely to hear a great deal more before their wishes were consummated. This is not a matter upon which the dental public at present could be considered well informed. They were the section of the community most closely concerned, and if they were to regard their own interests it was necessary that when the time came they should be able to declare their convictions with no uncertain sound.

It had been constantly said if the licentiate in dental surgery craves for distinction, why not let him take a medical or surgical degree in addition—admirable advice in its way, but undoubtedly the dental student in obtaining it acquires a vast amount of knowledge which is not germane and which does not make him more proficient. In these days of specialization a man who desires to be distinguished above his fellows in any calling must concentrate all his energies and focus them upon the studies which are most directly concerned.

We are very heavily indebted to the Senates of the Birmingham University for having led the way and instituted a degree in dentistry, and it would be very interesting to learn the reasons that prevailed upon them to adopt the particular course they had given out. The whole of the British educated public is familiar with the titles B. Sc. or D. Sc., which conveys at once the impression that the bearer has proved himself more than merely proficient in some special subject.

When the time comes care should be taken to ensure that the candidate is proficient in those arts which are essential to the successful practice of their every-day work. No one would deny but that their work was in the main a handicraft, and upon his skill as a handicraftsman the welfare of his patients and his own success depended. This was so manifestly obvious that it seemed a platitude to repeat, but yet he thought it very necessary, as, in his opinion, the standard of practical work was not proportionately equal to the standard of scientific knowledge at present demanded by the examiners of the candidates.

Mr. G. W. Watson, L.D.S., said: I have taken an interest in this matter for some years, and at the time the University Commission was sitting was in favor of making representations to the commission in respect to a higher dental degree, but got little or no support, although it would have been easier than now to have got our wishes carried out. There is no reason why we should not have a B.Sc. or D.Sc. in dental surgery as well as in any of the other sciences, and it seems to me it would not require very great elaboration of the present D.Sc. curriculum to suit our purpose, as nearly all the subjects accord with what is required, viz: anatomy, chemistry, physiology, etc.

At present it is only by taking the full medical curriculum that a higher status can be obtained, and a great many of the classes attended are of little or no use to the dental practitioner afterward, whereas by the method now advocated the energies of the student could be applied more especially to the subjects bearing on dental surgery as a science and profession.

Mr. H. B. Ezard, L.D.S., remarked on the desirability of every dental student having a better general education than the preliminary examination demanded. His ideal was, he feared, not possible of realization, but he would like to see every student take the M.A. degree before beginning his special studies. The advantage of an academic career crowned by an arts degree were in after life very great. He quoted an instance in which a candidate for a professorial chair, though admittedly excellently well qualified for the post he sought, was passed over because he had no degree.

Mr. J. Gordon Shiach, L.D.S., said: In arranging the curriculum for a university degree in dental surgery it must first be decided whether or not that degree is to compete with the L.D.S. diploma. If this be not the case and if the intention be to give an advanced dental degree, which would be a proof of scientific study of dentistry and of kindred subjects which would have a bearing upon any branch of the profession, then the first point to be noted is that there be no attempt to overlap the subjects required for the L.D.S. diploma. It seems to me that in these days what we want is to have the practical side of our students' training more thoroughly attended to, but while making such a statement, I do not deny the desirability of having a higher degree for a certain section, who may thereby be put in the way of not only advancing their own interests, but what is of far greater moment, the interests of the profession at large. If, instead of such a degree being given for a further examination upon subjects bearing upon the every-day practice of the general medical practitioner, it were given for such science subjects as would enable a student to go deeper into the study of his specialty, it would become a blessing to the development of our profession, but as the curriculum has been set forth by the Birmingham University, it appears to me to be little else than a mixture of the present dental and general medical curriculum, with the weak parts of the former emphasized, in so far as it ignores the dental hospital teaching and replaces it with that of the "Dental Department of a General Hospital." But with all this talk about higher teaching and higher degrees, are we not beginning at the wrong end? Are we not apt to forget the practical side of our profession? Are we not too much inclined to belittle the training of our apprentices in the mechanical department, and do we not forget too readily that it is really in the workroom that the skillful operator is cradled? This, however, by the way. Returning to the actual subject at hand, those in authority should be reminded that it is dentistry, not medical practice, which such a degree is intended to develop.

Mr. F. T. Turnbull, L.R.C.P. and S.L.D.S., said: I must say that this suggestion, that when a university degree does come (as it no doubt will in the near future), it should take the form of a science degree, was to me both a new and acceptable proposition. While granting that the L.D.S. diploma is an excellent qualification and all sufficient as a license to practice, there are, and always will be, certain students who are prepared to do more work than is actually required of them. Naturally, these men desire some recognition of their extra labors.

In the past the goal aimed at has been a medical diploma or degree. Now, although this is a desirable addition, and one which may bring certain advantages, there is no doubt that a considerable part of the time is spent in acquiring knowledge of certain subjects which, even if it be remembered, will not be of practical assistance to the dental surgeon in his daily work. This objection, however, does not only apply to a medical course, as there are also, in my opinion, certain portions in the dental curriculum which might with advantage be eliminated or curtailed, and more important subjects added and others extended. A degree, therefore, such as sketched by Dr. Guy, which would denote a higher standard of the strictly dental subjects than that expected or obtained in the L.D.S. diploma would, I think, be a distinct advantage to the dental profession.

Mr. Ezard has just mentioned the case of the aspirant to professorial honors who was barred, as he lacked the M.A. degree, and has suggested an "arts" degree as the suitable hall-mark of academic distinction. On this point I can not agree with him. The arts degree is a most desirable one to have, and one we would all like to possess, but it is asking too much from the student to take on an extra three or four years to an already long and arduous course.

In addition to this, the years spent in attaining the arts degree are, in my opinion, the best for acquiring the rudiments of a manipulative training.

Mr. R. Lindsay, L.D.S., said: That there is a demand on the part of the dental profession for some higher distinction than the license must be apparent to all. The fact that a large number of our students go to the trouble and expense of obtaining an additional medical qualification is proof of this. It is interesting to hear, as we have just heard, from a gentleman who has gone through this trouble and expense that as far as practical benefit is concerned the time and the money so expended are largely wasted. Here, then, is the point as matters stand at present. The only possible distinction for the aspiring dentist is the taking of a medical qualification, which does not make him a better dentist—it may even make him a worse one by withdrawing him at the critical period in his professional training from the practical work wherein almost solely his true interest lies. Therefore, we turn with interest and expectation to this question of a degree in dentistry in the hope that there may be found the beginning of a more rational state of things. If this hope is to be in any measure realized it can only be by bringing to bear upon the matter the serious thought and influence of the dental profession as a whole. And I can conceive no better action, as regards Scotland at least, than that this society, in conjunction with the Scottish Branch of the British Dental Association, should take steps to direct the professional thought to this subject and to elicit the professional opinion.

With the principles laid down in Dr. Guy's suggestions concerning the lines of action there has been, I note, a large agreement, and certainly any means whereby the object in view may be attained without the further increase in the number of different degrees, is worthy of sympathetic consideration. The association of dentistry with the widely-known and honored B.Sc. could only result in good to the profession. And again, that the license should remain the sole right to practice dentistry with the degree as an academic distinction superadded bearing no such right commends itself to us for its very simplicity, but it must not be supposed that such a concession carries with it any approval of the present conditions attached to the obtaining of the dental license, and it may be seriously questioned whether it is advisable to add to our educational fabric the superstructure of a degree while the foundation is in such unsatisfactory condition and so clamant for alteration and repairs. Neither upon the question of the nature of the degree to be sought nor upon the question of its being registrable as a qualification to practice, is opinion in the profession so harmonious as it appears to be in this meeting, and I reserve my judgment on both of these points for further consideration.

MR. J. DOUGLAS LOGAN, L.D.S., said: A higher degree for dental surgeons is both desirable and necessary. I agree with him that the L.D.S. diploma is quite a useful license for general practice, the curriculum embraces as much general medicine and surgery as any dental surgeon can make use of, probably more, and the license should therefore stand. The tendency of dental work is certainly not in the direction of medicine and surgery, and Dr. Guy shows a wonderful appreciation of the fact in calling for what everyone must allow to be a step in the right direction. Allow me to remind Dr. Guy, however, that it will take some time and much clamoring to rouse the university in this matter, but once that is done, there can be no doubt that some such scheme as he proposes will be a startlingly progressive one.

Mr. J. Wolston Watt, L.D.S., said: Without a more careful study of the contents of the paper, I can not say more than that the plan seems a very good one, if only the university will think so too, and adopt this suggestion. I must say, when I read the title of the paper, I concluded that it would advocate a separate university course of dental study, just as a prospective medical student has before him the option of taking his course of study at the university, and at the end obtaining a degree; or taking out the "extra-mural" curriculum, receiving as a result a "license." At present we dentists have to be content with the "license" as the result of our five to seven years' dental study, whereas the medical student can obtain the degree of M.D. in six or seven. However, I sincerely hope that this subject will again be brought up for discussion, after we all have had time to carefully study the paper, and form more definite opinions on the subject.

Dr. Fenwick said: I do not know that I am entitled to speak on the question of a degree for dental practitioners, being only a dental student myself, but if you will allow me as a general medical practitioner of some dozen years standing, and a prospective dentist, I will be pleased to give you my views on the subject.

My experience of the average dental student is, that the L.D.S. examination is about as much as he can manage, he has no great hankering after a higher degree in dentistry, if he got more gold filling, porcelain inlays and crowns to do, it would, in my opinion, be a surer way to earn his bread and butter. Again, by the time a man has got his L.D.S. the man who pays for his training usually thinks it time the money drain stopped and the money earning commenced.

Mr. GUY said he was agreeably surprised to find such a unanimity of opinion in support of this proposal, which he had purposely refrained from elaborating over much, as he desired rather to elicit the views of a widely-representative gathering of dentists on the general principle than to provoke a discussion on details. He thought that the provision of degrees in science would itself be a sufficient inducement to the best of their students to try for them, and would perhaps attract good men to the profession. It was hopeless to expect many men to take the M.A. degree before beginning the study of dentistry, though he admitted it would be a counsel of perfection. With regard to Mr. Lindsay's charge that he had changed his views on this matter, his withers were unwrung. In the paper from which Mr. Lindsay quoted, in speaking of a higher qualification, he had said that the higher surgical diplomas afforded all that was required in that way. To that view he still adhered, and he hoped he had made it sufficiently plain that the degree he desired to see instituted must not be in any sense of the word a qualification, either higher or lower, but purely an academic distinction. He had, in short, no desire that the university should usurp the function of licensing bodies. He thanked the society for the reception it had accorded to, and the interest it had shown in his paper. —*Dental Record.*

